# Miniscrew‐Supported Palatal Expansion in Adults: A Clinical Case Report

**DOI:** 10.1155/crid/3434769

**Published:** 2026-01-24

**Authors:** Enrique Miranda-Astocondor, Franz Tito Coronel-Zubiate, Betsy Quispe-Quispe, Ruben Aguirre-Ipenza, Antonio Diaz-Caballero

**Affiliations:** ^1^ Faculty of Dentistry, National University Federico Villarreal, Lima, Peru; ^2^ Faculty of Health Sciences, School of Stomatology, National University Toribio Rodríguez de Mendoza, Amazonas, Peru; ^3^ Faculty of Dentistry, National University del Altiplano, Puno, Peru; ^4^ Faculty of Health Sciences, University Continental, Lima, Peru; ^5^ Faculty of Dentistry, University of Cartagena, Cartagena, Colombia, unicartagena.edu.co

**Keywords:** case reports, miniscrew-assisted maxillary expansion, orthodontic, orthodontic appliance, palatal expansion technique

## Abstract

Maxillary transverse deficiency is a common issue in orthodontic clinics and presents a treatment challenge in adults due to increased resistance to midpalatal suture separation. Miniscrew‐assisted rapid palatal expansion (MARPE) has become an effective alternative by providing skeletal anchorage and minimizing side effects on teeth; however, little has been reported about its impact on underlying facial soft tissues. This case report follows the CARE guidelines for case reports and describes the clinical management of a young adult with maxillary transverse deformity treated with MARPE and corticopuncture, planned using tomography. The activation was performed gradually, under clinical and tomographic monitoring. The tomography showed transverse improvement with a parallel opening of the midpalatal suture of about 5 mm and no signs of adverse effects on adjacent teeth. Additionally, a 3D facial reconstruction revealed slight facial changes at the frontal level that were not clinically significant. It is concluded that MARPE expansion therapy was an effective and safe alternative for treating maxillary transverse deficiency in the adult patient in this case, with minimal impact on dental and facial structures.

## 1. Introduction

Transverse maxillary deformity is a common problem in orthodontic practices, with reported frequencies ranging from 9% to 23% of cases [1]. Clinically, it manifests as unilateral or bilateral posterior crossbite, although it can also occur without this condition, in cases with dental compensations observed, such as a narrow smile or a Wilson curve accentuated by the buccal inclination of the upper posterior teeth and lingual inclination of the lower posterior teeth [2, 3]. The treatment of choice in these cases is rapid maxillary expansion (RME), with over a hundred years of reports in orthodontics [2, 4–6].

Traditionally, RME is tooth‐supported, which leads to unwanted side effects such as tooth tilting, root resorption, marginal bone loss, and decreased buccal bone thickness [7, 8]. To control these undesirable effects, miniscrew‐assisted rapid palatal expansion (MARPE), which incorporates skeletal anchorage through temporary anchorage devices, has been proposed [9]. This approach has demonstrated significant improvements in skeletal, dentoalveolar, and periodontal effects [10]. Furthermore, several studies report that the opening pattern obtained with MARPE is less triangular than that of tooth‐anchored ERM (SD‐ERM), which has been associated with greater long‐term skeletal stability [11].

Despite favorable evidence of its efficacy and clinical benefits, the MARPE technique is not yet widely implemented, possibly due to a lack of proper diagnosis, a lack of familiarity with the application protocol, or its higher cost compared to conventional devices such as the Hyrax‐type expander. In addition to the above, reports do not typically compare the facial effects produced in patients undergoing MARPE, even though the skeletal changes produced could be reflected in the underlying tissues. However, even considering the current importance of aesthetics, their evaluation is important.

This report describes the diagnosis, procedure, and clinical course of a patient with dentoalveolarly compensated transverse maxillary deficiency treated with MARPE, detailing the work protocol and the results obtained at the skeletal, dentoalveolar, and facial levels.

## 2. Case Report

A 22‐year‐old female patient came to the clinic for orthodontic treatment, stating that she “wanted to improve her bite.” Her medical history included a history of adenoid hypertrophy removal. Intraoral examination revealed a triangular maxillary arch with anterior crowding, a Class III left molar and canine relationship, 0.5 mm overjet, and 5% overbite. There was no posterior crossbite, but a Wilson curve accentuated by buccal inclination of the upper molars and lingual inclination of the lower molars. A 1‐mm right lower midline deviation was observed and a thin gingival phenotype (Figures 1a, 1b, 1c, and 1e).

Figure 1Pretreatment images: (a) right lateral photograph, (b) frontal photograph, (c) left lateral photograph, (d) 3D facial reconstruction, (e) upper occlusal photograph, and (f) frontal tomographic section at the level of the first molars.

**Figure:**
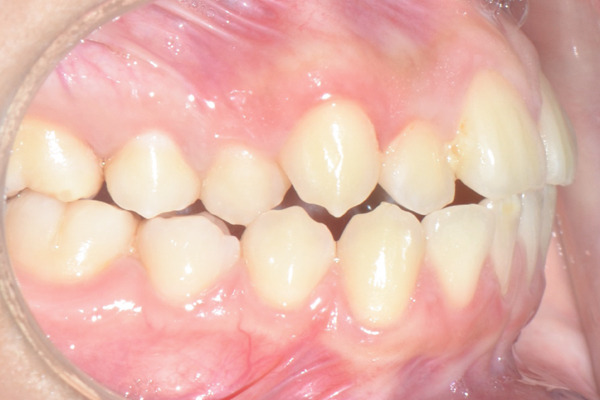
(a)

**Figure:**
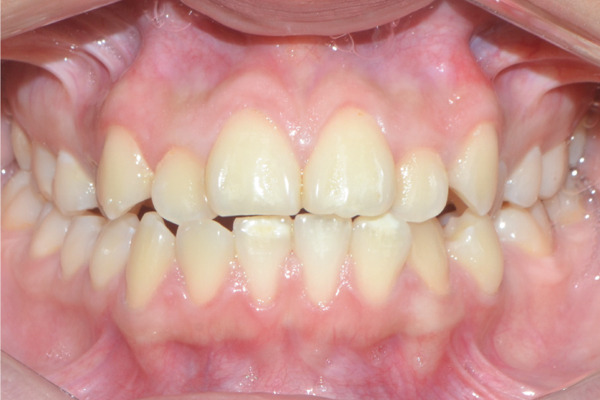
(b)

**Figure:**
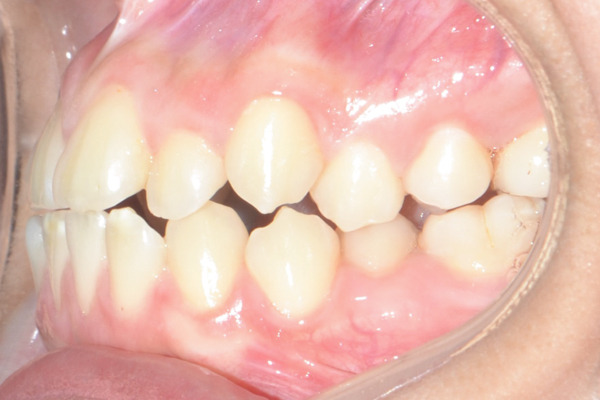
(c)

**Figure:**
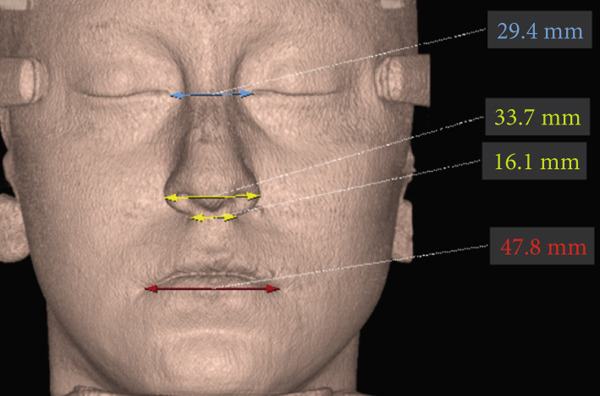
(d)

**Figure:**
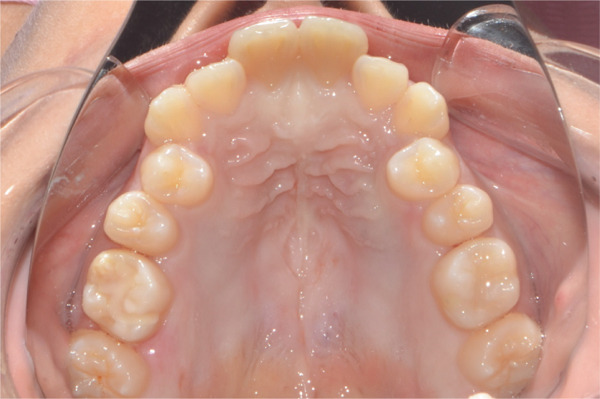
(e)

**Figure:**
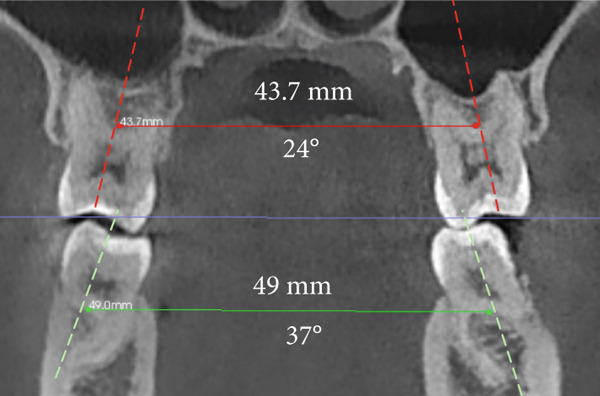
(f)

The imaging analysis consisted of a panoramic radiograph and a cone beam computed tomography (CBCT); in the tomography using the Xelis wiener software (Dental Viewer 1.0, Infinitt Healthcare, Seoul, South Korea), after its image standardization, the facial reconstruction was performed (Figure 1d) and the clinical distances at the frontal level of the nasal base, nasal interalar distance, ocular intercanthal distance, and labial intercommissural distance were measured. Skeletal measurements began by determining the transverse discrepancy of the maxilla with the Yonsei analysis [12], obtaining a maxillary deficiency of 5 mm (Figure 1f). The stage of sutural maturity was verified according to Anghelieri, and the patient was found to be in stage C [13] (Figure 2a). In addition, the height of the palate was measured, and a thin palate was found, taking measurements 3 mm posterior to the anterior palatine foramen, 4 mm anterior, and 2 mm posterior (Figure 2b). At the dental level, the angulation of the molars was measured, with the upper ones presenting an intermolar angulation towards the vestibular side of 24° and the lower ones an intermolar angulation towards the lingual side of 37° (Figure 1f).

Figure 2Images of measurements in CBCT: (a) axial cut, (b) sagittal cut in the midline, (c) frontal cut at the level of premolars, and (d) sagittal cut at 3 mm from the midline.

**Figure:**
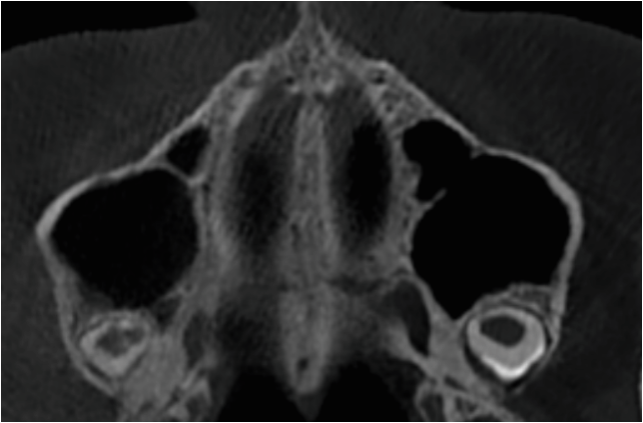
(a)

**Figure:**
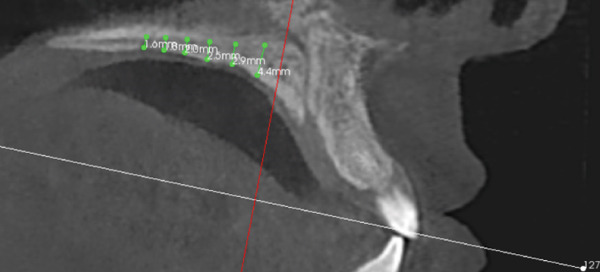
(b)

**Figure:**
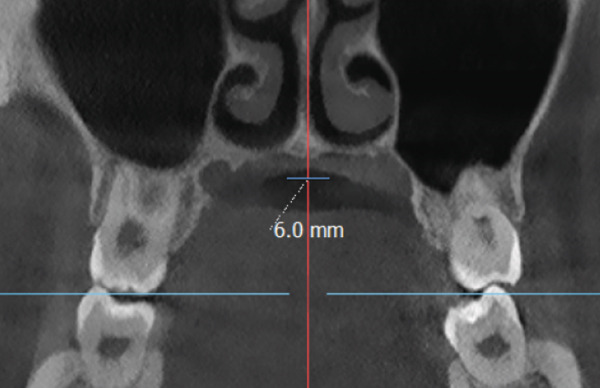
(c)

**Figure:**
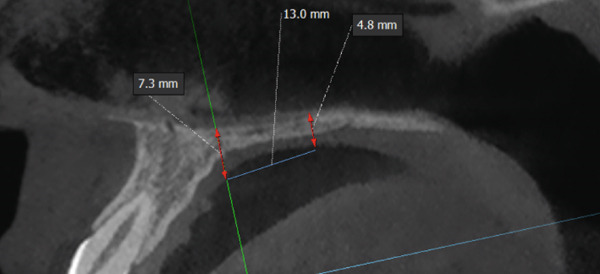
(d)

Due to the previously described characteristics of the sutural stage and the thin palate, and in order to avoid negative effects on the anchoring pieces due to the periodontal biotype, maxillary expansion with MARPE was chosen, to which we added the corticopuncture procedure in the palate to weaken the sutural interdigitation as described by Suzuki et al. [14], it is performed only once at the time of installing the appliances.

A disjunctor for 4 MARPE SL miniscrews (Peclab, Brazil) was selected in the working model; the anterior miniscrews were at the level of the second premolars and all paramedian to the midpalatal suture, in the well‐known safe T zone for screws in the palate [15], for better support, retention arms were made up to the first premolar. The miniscrews were selected based on tomographic measurements combining the reference position on the model and the distances between the screws of the selected expander (6 mm between anterior holes and 13 mm between anterior and posterior holes). The tomographic sections were made with these references, and the soft tissue distance and bone height were measured. Also, knowing that bicorticality is necessary for clinical performance, the bone height that should exceed the nasal cortex was increased by 1 mm. This gave the height of all the miniscrews: 5 mm for the posterior and 8 mm for the anterior (Figure 2c,d). Once the selection protocol was completed, the device was welded along the guidelines obtained from the tomography (Figure 3a,b). Once the appliance is ready, the clinical procedure begins aseptically with a 1‐min chlorhexidine rinse. An infiltrative anesthesia is administered anteriorly and posteriorly. For corticopuncture, a 6 × 1.5‐mm drill bit is used within a manual contra‐angle handpiece, and the puncture is made 1 mm below the nasal cortex, following the previously taken measurements (Figure 2b). The punctures are separated by 3 mm to the level of the second molar (Figure 3c). The MARPE appliance is cemented with glass ionomer, and the arms are joined to the premolars with fluid resin (Figure 3d). To install, the miniscrews are installed using a manual driver, a contra‐angle handpiece, and a long MARPE insertion wrench. They are inserted in a crisscross fashion, alternating anteriorly and posteriorly until a good fit is achieved without causing ischemia, but with a good insertion torque measured at 30 Ncm on the torque wrench. They are inserted in a crisscross manner, alternating anteriorly and posteriorly until a good fit is achieved without causing ischemia, but with a good insertion torque measured at 30 Ncm on the torque wrench. At the end of insertion, the patient reports no pain but does report an itchy nose, which is why she sneezed a few times (Figure 3e).

Figure 3Appliance installation: (a) appliance welded to the model, (b) MARPE, (c) corticoperforation, (d) appliance cementation, and (e) miniscrews inserted.

**Figure:**
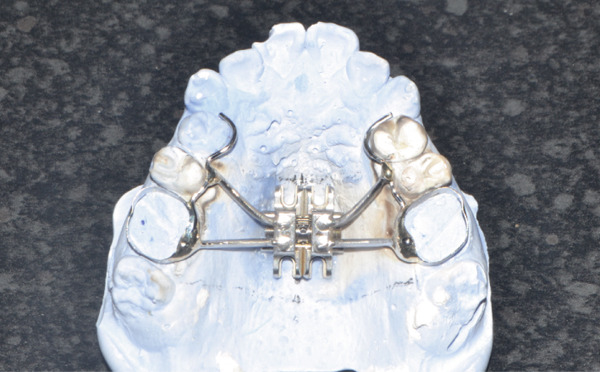
(a)

**Figure:**
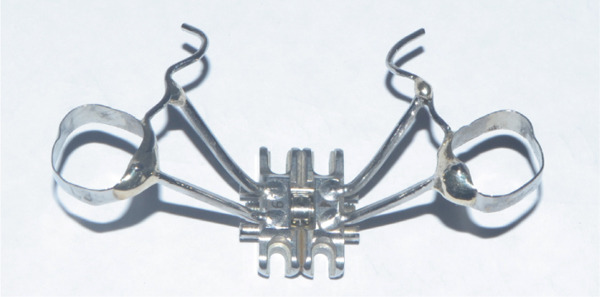
(b)

**Figure:**
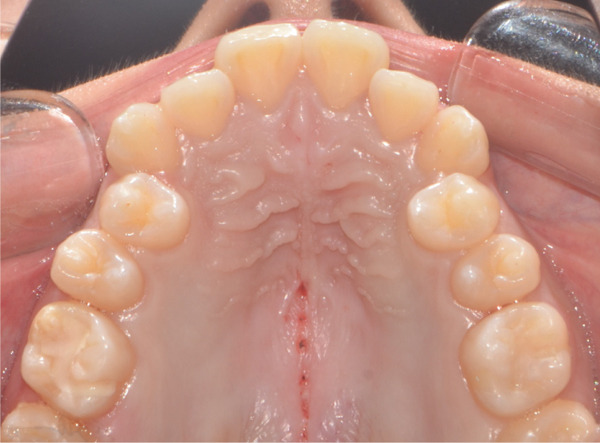
(c)

**Figure:**
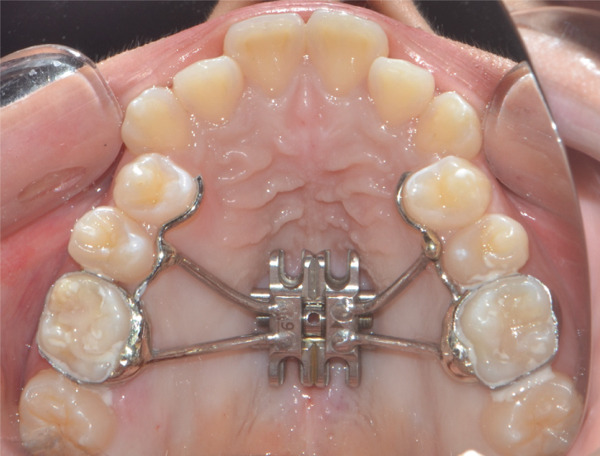
(d)

**Figure:**
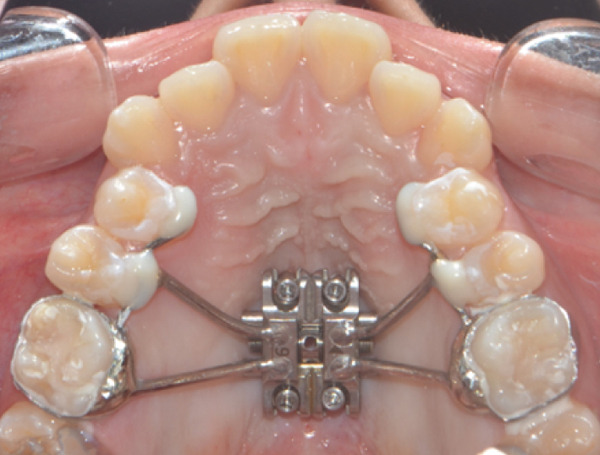
(e)

After installation, the patient was instructed on the necessary hygiene protocol using a soft brush, rinsing with 0.05% chlorhexidine, and using a cotton swab to cover the screw area with 0.12% chlorhexidine gel at night to prevent failure due to inflammation. She was scheduled to return in 1 week to begin activation to evaluate screw stability and to assess whether the corticopuncture was healing. After 1 week, the patient returned and was instructed to complete one turn of the expander screw in the morning.

Weekly check‐ups were performed; after 2 weeks, a median interincisal diastema was evident. After 1 month of continuous activation, the palatal cusps of the upper molars reached contact with the buccal cusps of the lower molars (Figures 4a, 4b, and 4c). During this check‐up, the patient reported improved breathing and noticed a slight widening of the nasal alae. Given clinical signs of successful expansion and to complete the active phase, the expansion screw is sealed with fluid resin (Figure 4e).

Figure 4Immediate post‐expansion images: (a) right lateral photograph, (b) frontal photograph, (c) left lateral photograph, (d) 3D facial reconstruction, (e) upper occlusal photograph, and (f) frontal tomographic section at the level of the first molars.

**Figure:**
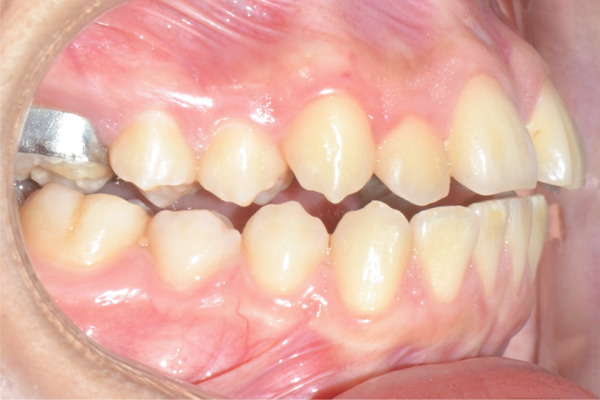
(a)

**Figure:**
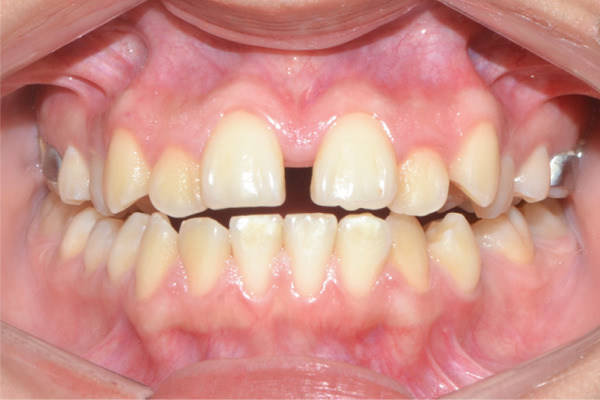
(b)

**Figure:**
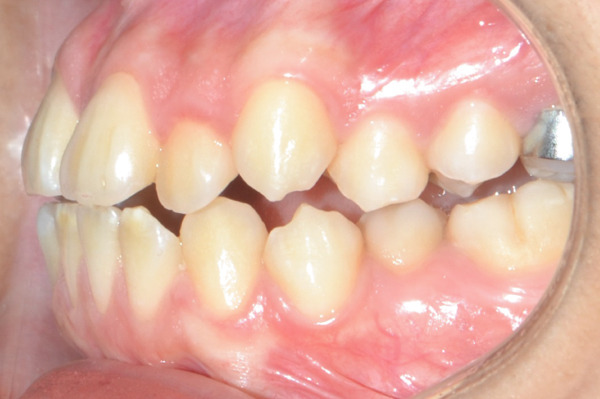
(c)

**Figure:**
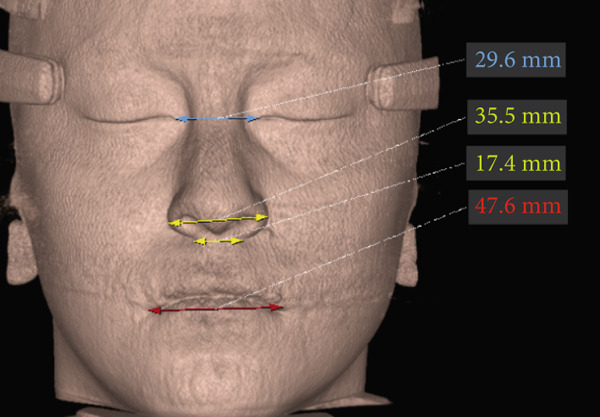
(d)

**Figure:**
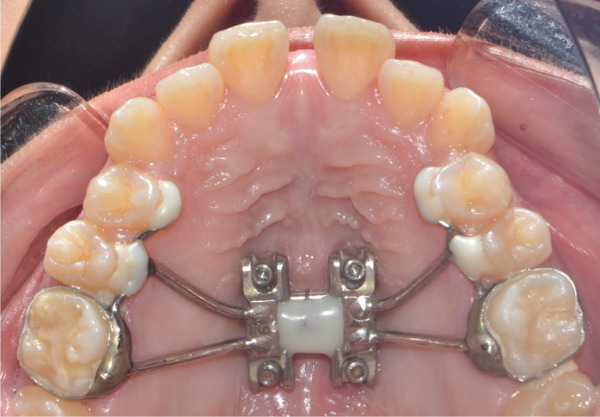
(e)

**Figure:**
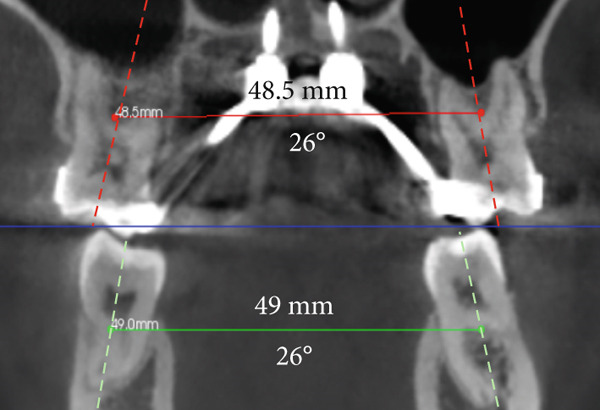
(f)

To verify, quantify, and compare the results obtained postexpansion, a TCHC is sent after sealing the MARPE (Table 1). The CTBC showed facial changes in the 3D facial reconstruction with measurements of the frontal distances of the nasal base at 17.4 mm, nasal interalar 35.5 mm, ocular intercanthal 29.6 mm, and labial intercommissural 47.6 mm (Figure 4D); the transverse distance of the upper maxilla was measured at 48.5 mm, mandibular transverse distance at 49 mm, upper molar inclinations at 26° towards the vestibular, and the inclination of lower molars at 26° towards the lingual (Figure 4f). The bicorticality of the miniscrews used was verified (Figure 5a), and the opening of the midpalatine suture showed a parallel opening pattern of approximately 5 mm along its entire length in both the axial and frontal views of the CBCT (Figure 5b,c).

**Table 1:** Comparison of values obtained pretreatment and postexpansion.

**Parameters**	**Pretreatment**	**Immediate postexpansion**	**Change**
Nasal base distance	16.1 mm	17.4 mm	+ 1.3 mm
Nasal interalar distance	33.7 mm	35.5 mm	+ 1.8 mm
Intercanthal ocular distance	29.4 mm	29.6 mm	+ 0.2 mm
Labial intercommissural distance	47.8 mm	47.6 mm	− 0.2 mm
Maxillary transverse distance	43.7 mm	48.5 mm	+ 4.8 mm
Mandibular transverse distance	49 mm	49 mm	0 mm
Anterior intersutural distance	0	5.1 mm	+ 5.1 mm
Posterior intersutural distance	0	5 mm	+ 5 mm
Angulation between upper molars	+ 24°	+ 26°	+ 2°
Angulation between lower molars	− 37°	− 26°	+ 11°
Overjet	0.5 mm	1.5 mm	+ 1 mm
Overbite	5%	− 10%	− 15%

Figure 5Immediate postexpansion CBCT images: (a) full skull medium transparency, (b) axial section, and (c) frontal.

**Figure:**
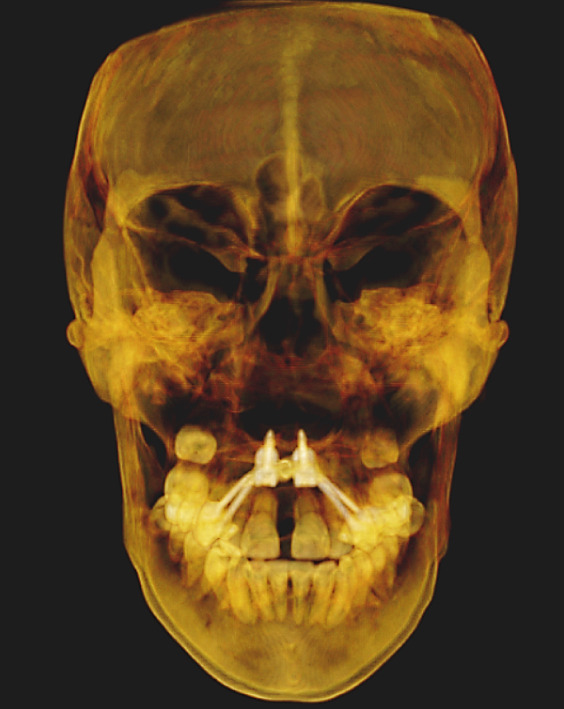
(a)

**Figure:**
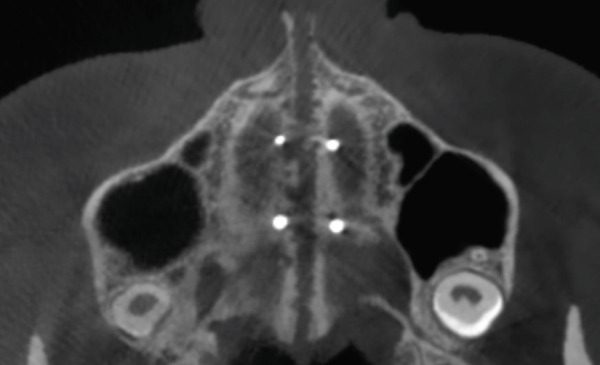
(b)

**Figure:**
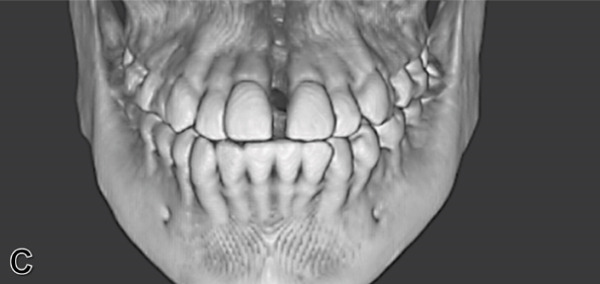
(c)

Once maxillary expansion was verified clinically and tomographically, the appliance was left in place. After 1 month, spontaneous closure of the midline diastema was observed, and the connecting arms to the teeth were removed, leaving the appliance installed as a transverse retainer, which would remain in place throughout the treatment. The upper fixed appliance with a 0.012 ^″^ NiTi archwire was then installed to begin alignment and leveling (Figures 6a, 6b, 6c, and 6d).

Figure 61 month postexpansion images: (a) upper occlusal, (b) frontal, (c) right side, and (d) left side.

**Figure:**
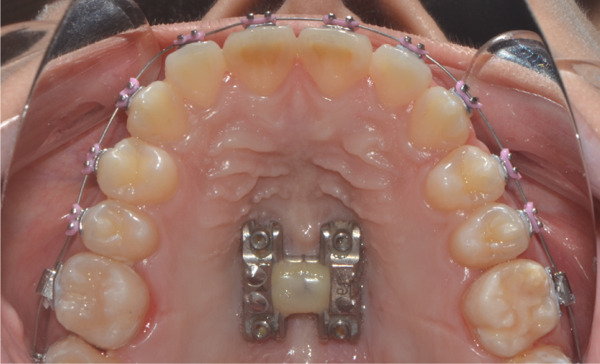
(a)

**Figure:**
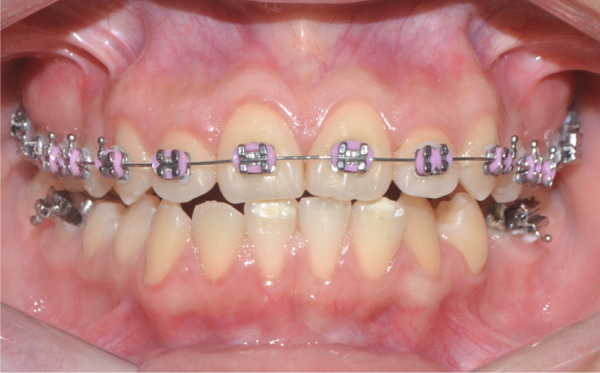
(b)

**Figure:**
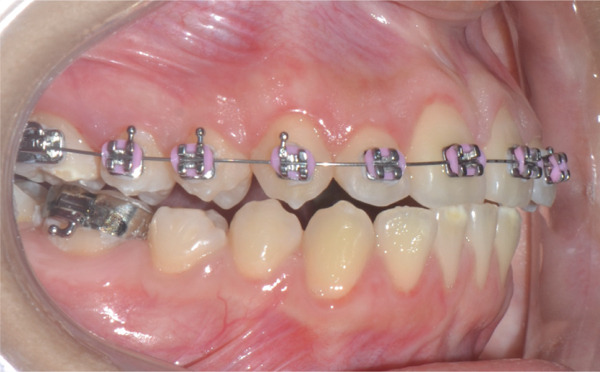
(c)

**Figure:**
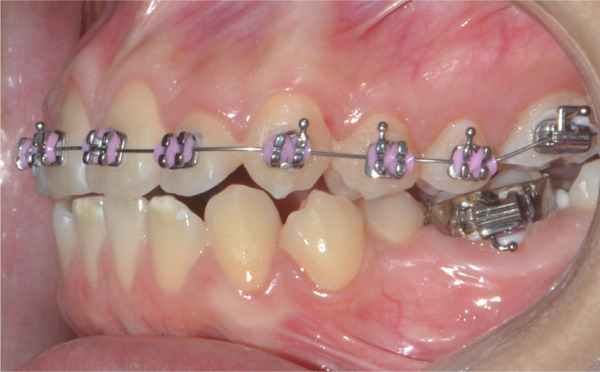
(d)

## 3. Discussion

This clinical case report is aimed at describing the diagnosis, procedure, and clinical evolution of a patient with dentoalveolarly compensated transverse maxillary deficiency, treated with maxillary expansion using MARPE. Skeletal and dentoalveolar results are reported, demonstrating its efficacy with minimal side effects. Furthermore, the facial effects at the frontal level are shown, assuming that changes in skeletal structures can reflect changes in adjacent facial tissues, which is of great importance in these times of high aesthetic demands for orthodontic patients [16].

When comparing soft tissue changes in 3D facial reconstruction using CBCT, a method validated as reliable and accurate for assessing facial changes according to previous studies [17], we observed a 1.3‐mm increase in the nasal base distance, a 1.8‐mm increase in the nasal interalar distance, and a 0.2‐mm increase in the intercanthal distance between the eyes. These measurements can be attributed to skeletal changes, with higher values occurring closer to the expansion zone. A 0.2‐mm decrease in the intercommissural distance between the lips was also noted. This is likely due to forced lip closure resulting from the increased lower vertical dimension caused by premature contact and mandibular rotation. Although these facial tissue changes are present, none are clinically significant, as the general public considers only changes greater than 2 mm to be relevant [18].

In the skeletal system, the purpose of MARPE therapy is to separate the midpalatal suture. This result was observed in both frontal and axial tomographic views (Figures 5a, 5b, and 5c), revealing a parallel opening along its entire length; this pattern differs from the triangular pattern achieved with dentally supported RME, which is attributed to the greater stability of the resulting expansion [7, 10, 11].

The maxillomandibular difference was quantified using the Yonsei index, and no posttreatment discrepancy was found. The success in the present clinical case, in addition to the corticopuncture performed, which helped weaken the palatal interdigitation, is attributed to the bicorticality obtained at the level of the floor of the nasal fossae, evidenced by CBCT (Figure 5a), as documented by Lee et al. [18].

Following suture removal, the absence of undesirable side effects on the anchor teeth was confirmed. A vestibular inclination of only 2° was observed in the first molars after therapy. A spontaneous improvement of 11° in the axial inclination of the lower molars was also observed (Figure 4e), which was also reported by Liao et al. with this therapy. [19].

While it is true that MARPE has already demonstrated benefits in hard tissues, airways, and control of dental effects, there are still aspects that require further investigation, such as the possible changes in underlying soft tissues that could affect the patient’s aesthetics. This report showed slight unfavorable aesthetic changes, which are consistent with studies indicating that MARPE produces minimal and clinically insignificant changes at the soft tissue level [20]. Further studies on this topic are needed, including different expansion amplitudes, facial patterns, and long‐term outcomes, to communicate and prevent patients from experiencing any facial changes anticipated with MARPE therapy.

This report, although successful, should not be generalized because it is a single case. However, it could be a starting point for establishing a protocol seeking greater efficacy and thus avoiding complications such as failure to achieve expansion or fractures in the maxilla when using MARPE [21].

## 4. Conclusion

The treatment of transverse maxillary deficiency with MARPE in an adult patient, presented in this report, demonstrated satisfactory skeletal results, as well as adequate control of adverse effects on the teeth, without negative facial repercussions. As clinicians, we can contribute to improved MARPE performance by maintaining a precise protocol for the selection of the expander screw and microscrews. We can also help weaken suture strength through controlled corticoperforations, if necessary, which further contributes to the clinical outcome, as shown in this case.

## Consent

The authors confirm that the patient’s informed consent was obtained for the inclusion of images and other clinical data in the publication. The patient is aware that every effort will be made to protect her identity, although absolute anonymity cannot be guaranteed.

## Conflicts of Interest

The authors declare no conflicts of interest.

## Author Contributions

E.M‐A.: methodology, data collection, writing original draft, and review. F.T.C‐Z.: writing original draft and review. B.Q‐Q.: writing original draft and review. R.A‐I.: resources and methodology and review. A.D‐C.: writing, review and editing, and supervision.

## Funding

The authors declare that no funds, grants, or other forms of financial support were received for the conduct of this study or the preparation of this case report.

## Data Availability

The data that support the findings of this study are available from the corresponding author upon reasonable request.
